# Irisin Promotes Osteogenesis by Modulating Oxidative Stress and Mitophagy through SIRT3 Signaling under Diabetic Conditions

**DOI:** 10.1155/2022/3319056

**Published:** 2022-10-10

**Authors:** Guangyue Li, Zixiang Jian, He Wang, Ling Xu, Tingwei Zhang, Jinlin Song

**Affiliations:** ^1^Chongqing Key Laboratory for Oral Diseases and Biomedical Sciences, Chongqing, China; ^2^Chongqing Municipal Key Laboratory of Oral Biomedical Engineering of Higher Education, Chongqing, China; ^3^College of Stomatology, Chongqing Medical University, Chongqing, China

## Abstract

Advanced glycation end products (AGEs) accumulate in the bone tissue of patients with diabetes mellitus, resulting in oxidative stress, poor bone healing, or regeneration. Irisin, a novel exercise-induced myokine, is involved in the regulation of bone metabolism. However, the effects of irisin on adipose-derived stem cell (ASC) osteogenic differentiation and bone healing under diabetic conditions remain poorly understood. ASCs were obtained from inguinal fat of Sprague-Dawley rats and treated with different concentrations of AGEs and irisin. Cell proliferation, apoptosis, and osteogenic differentiation abilities of ASCs were detected. To explore the regulatory role of sirtuin 3 (SIRT3), ASCs were transfected with lentivirus-mediated SIRT3 overexpression or knockdown vectors. Next, we investigated mitochondrial functions, mitophagy, and mitochondrial biogenesis in different groups. Moreover, SOD2 acetylation and potential signaling pathways were assessed. Additionally, a diabetic rat model was used to evaluate the effect of irisin on bone healing in calvarial critical-sized defects (CSDs) in vivo. Our results showed that irisin incubation mitigated the inhibitory effects of AGEs on ASCs by increasing cell viability and promoting osteogenesis. Moreover, irisin modulated mitochondrial membrane potential, intracellular ROS levels, mitochondrial O_2_^·−^ status, ATP generation, complex I and IV activities, mitophagy, and mitochondrial biogenesis via a SIRT3-mediated pathway under AGEs exposure. Furthermore, in calvarial CSDs of diabetic rats, transplantation of gels encapsulating irisin-pretreated ASCs along with irisin largely enhanced bone healing. These findings suggest that irisin attenuates AGE-induced ASC dysfunction through SIRT3-mediated maintenance of oxidative stress homeostasis and regulation of mitophagy and mitochondrial biogenesis. Thus, our studies shed new light on the role of irisin in promoting the ASC osteogenesis and targeting SIRT3 as a novel therapeutic intervention strategy for bone regeneration under diabetic conditions.

## 1. Introduction

Epidemiological investigations have revealed that an increasing number of patients are diagnosed with diabetes, among whom almost 90% have type 2 diabetes mellitus (T2DM) [1]. For this vast group of patients, bone defect regeneration remains a large challenge since they may have a high risk of impaired bone healing or regeneration [2]. According to previous studies, inhibited proliferation and decreased expression of osteogenesis-related transcription factors in osteoblasts (osteoprogenitors) largely contribute to delayed bone healing in T2DM [3]. Thus, finding strategies to improve impaired bone healing is crucial for T2DM patients with bone fracture or bone defects.

Recently, stem cell-based tissue engineering of bone has appeared to be a promising approach to restore bone defects [4]. A variety of mesenchymal stem cells (MSCs) have been employed to promote bone healing in diabetic patients, among which adipose-derived stem cells (ASCs) have attracted extensive attention from researchers. ASCs not only have potent proliferative capacity and multilineage differentiation potential but also can be isolated from multiple adipose tissues in large amounts and exhibit low immunogenicity features [5].

However, studies have indicated that diabetic microenvironments, namely, those with relatively high glucose levels and chronic inflammation, could affect the biological characteristics and osteogenic differentiation of MSCs [6]. Advanced glycation end products (AGEs) are commonly formed under chronic hyperglycemia, leading to abnormal protein, nucleic acid, and lipid functions [7]. Accumulating evidence has revealed that during aging and in diabetes, the formation and accumulation of AGEs accelerate and result in high oxidative stress conditions [8]. According to previous studies, reactive oxygen species (ROS) have been identified as a key player in many pathogenic processes associated with complications in DM patients [9]. Mitochondrial structural and functional integrity can be adversely impacted by oxidative stress [10], potentially perturbing autophagy machinery and promoting release of proinflammatory mediators and/or apoptotic cell death [11]. An additional study demonstrated that the deposition of AGEs within bone and adipose tissue is increased in diabetic mouse models and that AGEs could impair the osteogenic capacities of ASCs by increasing DNA methylation levels [12]. However, reliable and effective drugs or molecules that induce ASC osteogenesis and promote bone formation under hyperglycemic conditions have yet to be found.

In 2012, irisin was first discovered as a myokine that mediates a variety of effects of exercise in multiple tissues [13]. Exercise can induce the expression of peroxisome proliferator-activated receptor gamma coactivator 1*α* (PGC-1*α*), which subsequently stimulates an increase in the expression of fibronectin-type III domain-containing 5 (FNDC5), a transmembrane protein that is cleaved and secreted as a hormone-like peptide, irisin [14]. Although irisin was initially found to be involved in the browning of white adipose tissue, increasing evidence indicates that it plays a novel role in multiple metabolic syndromes as well as in bone metabolism [15]. In a group of postmenopausal women with low bone mass, irisin levels in the circulation closely correlated with bone mineral status and osteoporotic fracture risk [16], suggesting that irisin may act as a biochemical marker of bone mineral-related diseases. Further studies confirmed the potential effects of irisin on bone metabolism by indicating that irisin could not only prevent the inhibition of osteoblast differentiation under stimulated microgravity conditions but also enhance the osteogenic and chondrogenic differentiation of MSCs by modulating the Wnt/*β*-catenin signaling pathway [17–19]. However, the effect of irisin on cell injuries and ASC osteogenic differentiation under hyperglycemic conditions is not fully understood.

Sirtuin 3 (SIRT3), a member of the NAD^+^-dependent deacetylase family, is mainly located in mitochondria, is involved in enzyme activity regulation through deacetylation, and may have effects on mitochondrial functions and other cellular processes [20]. Previous findings have addressed the critical role of SIRT3 in modulating oxygen metabolism and eliminating intracellular ROS [21]. Moreover, growing evidence has indicated that SIRT3 contributes to bone metabolism in a protective way [22]. According to one study, SIRT3 expression was evidently elevated in the process of osteogenesis, while SIRT3 knockdown led to mitochondrial dysfunction and impaired osteogenic differentiation potential in osteoblasts [23]. Moreover, abnormal SIRT3-mediated mitophagy has been suggested to be closely linked with bone metabolism disorders, such as age-associated osteoporosis [24].

Herein, we hypothesized that irisin may exert favorable effects on ASCs in a diabetic microenvironment, probably through SIRT3-mediated intracellular mechanism. In the present study, we found that irisin plays a protective role in maintaining the intracellular homeostasis of ASCs under diabetic conditions via the SIRT3-mediated signaling pathway. In addition, a potential therapeutic effect of irisin-treated ASCs on calvarial critical-sized defect (CSD) regeneration under diabetic conditions was evaluated. By using diabetic rat models and modifying the SIRT3 gene with lentivirus vector transfection, we explored the effects of irisin on the proliferation and osteogenesis of ASCs in environments with AGEs and unveiled the underlying molecular mechanisms. Our studies revealed the role of irisin in osteogenesis and targeting SIRT3 as a novel therapeutic strategy for bone regeneration under diabetic conditions.

## 2. Materials and Methods

### 2.1. Materials/Chemicals and Antibodies

Bovine serum albumin (BSA) and advanced glycation end products (AGEs) were purchased from Biovision (San Francisco, USA). Recombinant irisin was obtained from Sigma-Aldrich (St. Louis, MO, USA). Cell culture medium *α*-MEM and fetal bovine serum were provided by HyClone (Pittsburgh, USA) and Gibco-Invitrogen (Grand Island, USA). Osteogenic and adipogenic induction media were obtained from Cyagen (Santa Clara, CA, USA). Annexin-V fluorescein isothiocyanate (FITC) apoptosis detection kit and JC-1 probe were obtained from Beyotime (Shanghai, China). Primary antibodies for Bax, SIRT3, LC3, Parkin, p62, and *β*-actin were provided by Cell Signaling Technology (Danvers, MA, USA); Bcl-2 and AIF antibodies were from Santa Cruz (Dallas, Texas, USA); PINK1, acetyl-SOD2-K68, and SOD2 antibodies were from Abcam (Cambridge, UK); and p-AMPK, t-AMPK, and PGC-1*α* antibodies were from Beyotime. And the secondary antibodies were provided by Thermo Fisher Scientific (Waltham, MA, USA). The hydrogel scaffolds used in calvarial defects were purchased from Sigma-Aldrich (HYSHP020, St. Louis, MO, USA) and prepared following the manufacturer's instructions.

### 2.2. Culture and Characterization of Isolated ASCs

All animal experiments in this study were performed in accordance with the Declaration of Helsinki and the National Institutes of Health Guide for the Care and Use of Laboratory Animals and were approved by the Medical Ethics Committee of the School of Stomatology, Chongqing Medical University (2020010). Ten Sprague-Dawley (SD) rats (male; 4-week-old) were purchased from the Animal Experimental Center of Chongqing Medical University. Euthanasia of the animals was performed with CO_2_ exposure. The rats in cages were exposed to CO_2_ at a flow rate of 5 l/min until one minute after breathing stops. After confirmation of euthanasia by decapitation, we obtained the inguinal fat pads under sterile conditions as previously described [25]. Briefly, adipose tissues were collected and finely cut into smaller pieces, then treated with 0.075% type I collagenase (Sigma-Aldrich, USA) for 30 minutes. After being centrifuged at 200 × g for 5 mins, cell pellets of ASCs were resuspended in growth medium (GM) consisting of *α*-MEM (Hyclone, USA), 10% fetal bovine serum (FBS), and 100 U/ml penicillin-streptomycin (Hyclone, USA). Cells were cultured in flasks under a standard humidified atmosphere of 5% CO_2_ at 37°C and subcultured (1 : 3) when reaching 70-80% confluence. Osteogenic and adipogenic differentiation potentials of third-passage ASCs were estimated. Furthermore, a commercial kit with FITC anti-rat antibodies (Cyagen), including CD29, CD44, and CD73 for mesenchymal stromal cell markers and CD34 and CD45 for hematopoietic cell markers, was used to detect cell surface markers of third-passage ASCs, following the manufacturer's instructions.

### 2.3. Cell Viability Assay

The cell counting kit-8 kit (CCK8, DOJINDO, Shanghai, China) was used to detect cell proliferation. Briefly, the third-passage ASCs were seeded in 96-well plates at a density of 5 × 10^3^ cells/well. After 24 h culture, ASCs were treated with 20, 40, and 80 *μ*g/ml AGE-BSA or 80 *μ*g/ml BSA control diluted in GM, for 24, 48, and 96 h, respectively. To estimate the effects of irisin on ASCs under AGE-induced environment, irisin was first reconstituted in ddH_2_O and prepared in GM with various concentrations of 1, 10,100, and 1000 ng/ml; then, ASCs were incubated with GM with different concentrations of irisin and 40 *μ*g/ml AGEs according to different groups, for 48 h and 96 h. The optical density (OD) values were read at the 450 nm wavelength.

### 2.4. Cell Apoptosis

Apoptosis was analyzed by flow cytometry using Annexin-V fluorescein isothiocyanate (FITC) and propidium iodide (PI) detection kit (Beyotime, China). Briefly, the third-passage ASCs were seeded in 6-well plates in GM at a density of 5 × 10^4^ cells/ml. After 24 h culture, ASCs were pretreated with 100 ng/ml irisin for 2 h and then treated with or without 40 *μ*g/ml AGEs for another 48 h in the presence of irisin. Then, cells were collected and incubated with a mixture containing Annexin V-FITC and PI for 20 min in the dark and detected on a BD Influx flow cytometer (BD Biosciences, San Jose, CA). The total apoptosis rate of ASCs was calculated as the sum of rates of cells observed in the lower-right quadrant (early-phase apoptotic cells) and the upper-right quadrant (late-phase apoptotic/necrotic cells).

### 2.5. Caspase-3 Activity

A caspase-3 activity assay kit (Beyotime, China) was used according to the manufacturer's protocol. Briefly, the third-passage ASCs were seeded in 96-well plates in GM at a density of 5 × 10^3^ cells/well. After 24 h culture, ASCs were pretreated with 100 ng/ml irisin for 2 h and then treated with or without 40 *μ*g/ml AGEs for another 48 h in the presence of irisin; ASCs were lysed and centrifuged. After 1 h incubation of 0.2 mM Ac-DEVD-pNA substrate, caspase-3 activity was measured by a microplate reader at 405 nm (Molecular Devices, USA).

### 2.6. Alkaline Phosphatase (ALP) and Alizarin Red Staining (ARS)

The third-passage ASCs were seeded into 6-well plates at a density of 5 × 10^4^ cells/ml. After culturing for 24 hours, GM was replaced by osteogenic differentiation medium (OM), which included GM supplemented with ascorbate (5 *μ*mol/l), *β*-glycerophosphate (10 mmol/l), and dexamethasone (100 nmol/l), as well as 40 *μ*g/ml AGEs along with various concentrations of irisin according to different groups. During the osteogenic differentiation period, OM with different concentrations of irisin along with 40 *μ*g/ml AGEs were changed every other two days. A BCIP/NBT ALP kit (Beyotime, China) was used to detect ALP on day 7 of osteogenic induction. ARS was carried out after 21 days of osteogenic induction in vitro. Images of stained cells were acquired under an inverted phase-contrast microscope. Stained mineralized nodules were dissolved with 10% cetylpyridinium chloride (Sigma-Aldrich, USA), and the OD value was measured at 570 nm.

### 2.7. Cell Transfections and RNA Silencing

Lentivirus-mediated SIRT3 overexpression (Lv-SIRT3) and lentivirus-mediated SIRT3 knockdown (Lv-shSIRT3) constructs were synthesized by Genechem (Shanghai, China). ASCs of third passage were cultured in 6-well plates in GM at an initial density of 1.2 × 10^6^/well. When the cells reached about 70% confluency, ASCs were transfected with the constructed lentivirus harboring rat SIRT3 shRNA, the SIRT3-coding sequence, or GFP-expressing control vector (Genechem, Shanghai, China) at a multiplicity of infection (MOI) of 20. After incubation with the above constructs for 8-12 h, the medium was replaced by GM for another 72 h culture. For the transient knockdown of PGC-1*α*, PGC-1*α*-siRNA and negative control-siRNA (NC-siRNA) were designed and synthesized by GenePharma Corporation (Shanghai, China), the transfection was performed by using LipoHigh reagent (Sangon Biotech, China), with LipoHigh : siRNA = 5 *μ*I : 100 pmol for each well of 6-well plates. Transfection efficiencies of SIRT3 and PGC-1*α* were confirmed with Western blot and real-time PCR.

### 2.8. RNA Isolation and Quantitative Polymerase Chain Reaction (qPCR) Analysis

Total RNA was extracted with RNAiso Plus Reagent (Takara, Beijing, China) according to their manufacturer's protocols. cDNA was generated by reverse transcribing total RNA with PrimeScript™ RT Master Mix (Takara) using 500 ng total RNA as a template. qPCR was subsequently performed by a CFX96 Touch™ Real-Time System (Bio-Rad, USA) using TB Green™ Premix Ex Taq™ II (Takara). In order to generate a relative expression ratio, the mRNA level of the gene of interest was normalized to the geometric average mRNA level of the *β*-actin housekeeping gene (Takara). The data analysis was performed using the 2^-*ΔΔ*Ct^ method and expressed as fold change compared to respective controls. The primer sequences are listed in Table 1.

### 2.9. Mitochondrial Membrane Potential (MMP), Intracellular Reactive Oxygen Species (ROS), Mitochondrial O_2_^·−^, ATP Production, and Mitochondrial Complex Assays

For the detection of MMP, intracellular ROS, and mitochondrial O_2_^·−^ within ASCs in different groups, third-passage ASCs were seeded onto four-chamber 35 mm glass bottom dishes (Cellvis, CA, USA) at 40-60% confluency. After 24 h culture in GM, ASCs were pretreated with 100 ng/ml irisin for 2 h and then treated with or without 40 *μ*g/ml AGEs for another 48 h in the presence of irisin. A lipophilic probe, JC-1 (Beyotime, China), was used to detect MMP (*Δψ*m). Different groups of ASCs were incubated with JC-1 working solution for 20 min at 37°C, washed twice with washing solution, and maintained in GM. The fluorescence intensity was determined by a confocal laser scanning microscope (CLSM, Leica, Germany). The quantitative analysis of the red/green fluorescence signal was detected, and ImageJ software (version1.8.0, NIH, USA) was used for quantification and calculation. The *Δψ*m was represented as the ratio of red to green fluorescence intensity. To analyze the intracellular oxidative stress levels, a Reactive Oxygen Species Assay Kit (Beyotime, China) was used. Briefly, cells were counterstained with Hoechst 33342 Dye (Beyotime, China) for 10 min in the dark and washed, followed by observation using CLSM. The fluorescence values were read at an excitation wavelength of 488 nm and emission wavelength of 525 nm for DCF and excitation wavelength of 350 nm and the emission wavelength of 461 nm for Hoechst 33342. The ImageJ software was used for quantitative analysis of the green fluorescence signals. To assess mitochondrial O_2_^·−^ status, we used MitoSOX Red probe (Cat #M36008, Invitrogen, Carlsbad, USA). Cells were treated as indicated and then incubated with 5 *μ*M working solution of MitoSOX Red and 200 nM MitoTracker Green (Cat # C1048, Beyotime, China) in the dark at 37°C for 15 min. Cells were visualized by CLSM, with the excitation wavelength of 510 nm and the emission wavelength of 580 nm for MitoSOX Red and excitation wavelength of 490 nm and the emission wavelength of 516 nm for MitoTracker Green, respectively. ATP production of ASCs was investigated using ATP Assay Kits (Beyotime, China), according to the manufacturer's instructions. Third-passage ASCs were seeded in 6-well plates at a density of 5 × 10^5^/ml. After 24 h culture in GM, ASCs were pretreated with 100 ng/ml irisin for 2 h and then treated with or without 40 *μ*g/ml AGEs for another 48 h in the presence of irisin. Then, 1 × 10^6^ ASCs of each group were lysed in ATP Lysis Buffer and centrifuged at 12000 × g at 4°C for 5 min. After gentle mixing of sample and ATP reaction buffer, the readings were taken with the luminometer (Molecular Devices, USA). ATP contents were calculated by using an ATP standard curve. To evaluate the activities of complexes I and IV in mitochondrial electron transport chain, mitochondria were firstly isolated from ASCs using the Cell Mitochondria Isolation Kit (Beyotime, China) as previously described [26]. The activities of the mitochondrial complex I or IV were determined by commercial kits available from Solarbio (Beijing, China). Mitochondrial complex activity was expressed as nanomole per minute per milligram of protein.

### 2.10. Antioxidative Enzyme Activity Detection

The enzyme activities of superoxide dismutase 2 (SOD2), catalase (CAT), and glutathione peroxidase (GSH-Px) were assessed by using commercial assay kits (Beyotime, China) according to the manufacturers' instructions, respectively.

### 2.11. Protein Extraction and Western Blot (WB)

ASCs were washed with PBS and lysed in RIPA buffer (Beyotime, China) supplemented with 1% PMSF and phosphatase inhibitor cocktail (Beyotime, China) on ice, and they were subsequently lysed by ultrasound. After centrifugation at 12000 × g at 4°C for 10 min, the supernatant containing the total protein was obtained. Equal amounts of total lysate were subjected to 12% SDS-PAGE, then transferred to a PVDF membrane (Solarbio, China), blocked with 5% nonfat milk, then incubated with primary antibodies at 4°C overnight, and further incubated with secondary antibodies (Thermo Fisher Scientific Inc, USA) at room temperature for 2 h before being visualized by a hypersensitive ECL chemiluminescence kit (Beyotime, China). Quantification of WB data was performed using ImageJ software (National Institutes of Health, USA), and the expression of target proteins was normalized to *β*-actin. The first antibodies used in the experiment are shown as follows: Bcl-2 (1 : 1000, Santa Cruz, USA), AIF (1 : 1000, Santa Cruz, USA), Bax, SIRT3, Parkin, LC3, p62, *β*-actin (1 : 1000, Cell Signaling Technology, USA), PINK1, acetyl-SOD2-K68 and SOD2 (1 : 1000, Abcam, UK), PGC-1*α*, p-AMPK, and t-AMPK (1 : 1000, Beyotime, China).

### 2.12. Immunofluorescence Imaging

To determine the mitophagy status within ASCs in different groups, third-passage ASCs were seeded onto four-chamber 35 mm glass bottom dishes at 40-60% confluency. After 24 h culture in GM, ASCs were pretreated with 100 ng/ml irisin for 2 h and then treated with or without 40 *μ*g/ml AGEs for another 48 h in the presence of irisin. ASCs were stained with MitoTracker Red (Beyotime, China) in the dark at 37°C for 30 min; then, ASCs of each group were fixed with 4% PFA for 15 min and permeabilized with 0.3% Triton X-100 for 10 min at room temperature, followed by blocking with 5% goat serum and 0.3% Triton X-100 for 60 min, and further incubated with anti-LC3 (1 : 100 dilution) overnight at 4°C. After washing with PBS, the cells were incubated for 1 h at room temperature with an Alexa Fluor 488-conjugated secondary antibody (1 : 500 dilution; Beyotime, Shanghai, China). Finally, the cells were washed with PBS and the nuclei were counterstained with 4′, 6′-diamidino-2-phenylindole (DAPI) for 5 min at room temperature. Images of the stained cells were acquired under CLSM.

### 2.13. Induction of Diabetes in Rats

Rats were fed with high-fat diet (HFD) consisting of fuel energy of 5.1 Kcal/g, comprising 60% calories from fat, 20% from protein, and 20% from carbohydrate (Boaigang, Beijing, China). After 4-week HFD feeding, rats were given a single intraperitoneal injection of streptozotocin (STZ) (40 mg/kg, Sigma, USA) to induce T2DM models as previously described [27]. Three days after STZ injection, the rats with blood glucose concentration greater than 16.7 mmol/l were selected as diabetic rats. After two weeks of steady blood glucose concentrations more than 16.7 mmol/l, the diabetes model was considered successfully induced.

### 2.14. Generation of Calvarial Critical-Sized Defect (CSD) Animal Model

After establishing the diabetic models, 30 male SD rats (10-11 weeks old) from Medical University were divided into the following five groups (*n* = 6): (1) control (empty defect), (2) blank gels, (3) gels encapsulating 5 × 10^3^ ASCs, (4) gels encapsulating 100 ng/ml irisin, and (5) gels encapsulating 5 × 10^3^ ASCs (preincubated in 100 ng/ml irisin for 2 h) and 100 ng/ml irisin. The hydrogels we used in experiments were the commercial kit as HyStem™-HP Hydrogel Scaffold obtained from Sigma-Aldrich (Cat No. HYSHP020), which are suitable for stem cell culture and allow slow release of incorporated growth factors. Gels of different groups were prepared following the manufacturer's instructions. Briefly, under aseptic conditions, HyStem-HP and Gelin-S were dissolved in degassed water by shaking for 30 min to make 1x stock solutions. Then, the above two stock solutions were mixed, and suitable amounts of irisin or pretreated ASCs were added into mixture according to experiment protocols. Then, the hydrogels were formed by combining the above mixture and 1x Extralink 2 stock solution at the proportion of 4 : 1. According to our previous published study [28], all animals were anesthetized and two 5 mm critical-sized calvarial defects were carefully made on each side of the cranium using a trephine drill, avoiding damage to the dura mater. During the drilling, the surgical area was flushed with normal saline. The gels were implanted in the defects or the defects were left empty as a control according to the above groups. After operation, the incision was sutured layer by layer. All animals survived after surgery and fully recovered after 24 h.

### 2.15. Microcomputed Tomography (Micro-CT)

At 8 weeks postsurgery, euthanasia of the animals was performed with CO_2_ exposure. The rats in cages were exposed to CO_2_ at flow rate of 5 l/min until one minute after breathing stops. After confirmation of euthanasia by decapitation, we harvested the skulls and fixed them in 10% neutral buffered formalin and scanned them by a micro-CT (Viva CT40, SCANCO Medical, Switzerland) at a resolution of 17.5 mm. The defect region was then identified by a cylindrical contour as a region of interest (ROI), and microstructural parameters of ROI including bone volume/total volume (BV/TV) and bone mineral density (BMD) were calculated using SCANCO analysis software.

### 2.16. Paraffin Sections and Staining

Specimens were decalcified in 10% EDTA for at least 2 months and then dehydrated and embedded in paraffin. Serial sections (5 *μ*m thickness) were prepared along the coronal plane and stained with hematoxylin and eosin (HE) and Masson's Trichrome (MTC) stain kit (Solarbio, Beijing, China) for histological analysis. Further, specimens were also permeabilized in 0.3% Triton X-100 and then blocked in 5% normal goat serum, incubated overnight with primary antibodies against osteocalcin (OCN) (1 : 200) and Collagen I (Col1) (1 : 200) (Bioss, Beijing, China) at 4°C, and stained with HRP-conjugated secondary antibodies (ZSJQ-BIO, Beijing, China). Diaminobenzidine (DAB) was used as a substrate for color development. Images of stained sections were obtained using a BX41 microscope (Olympus, Japan).

### 2.17. Statistical Analysis

All the experiments were repeated at least three times, and results are presented as mean ± SEM. An unpaired Student *t*-test was used when two independent groups were analyzed. For multiple comparisons, one-way analysis of variance (ANOVA) was performed with a Tukey post hoc test. Analyses were performed using GraphPad Prism software (version 8.0.2, USA). A *P* value of less than 0.05 (^∗^*P* < 0.05, ^∗∗^*P* < 0.01, ^∗∗∗^*P* < 0.001, and ^∗∗∗∗^*P* < 0.0001) was considered as statistically significant difference.

## 3. Results

### 3.1. Irisin Mitigates AGE-Induced Cell Injury in ASCs

**Figure 1:**
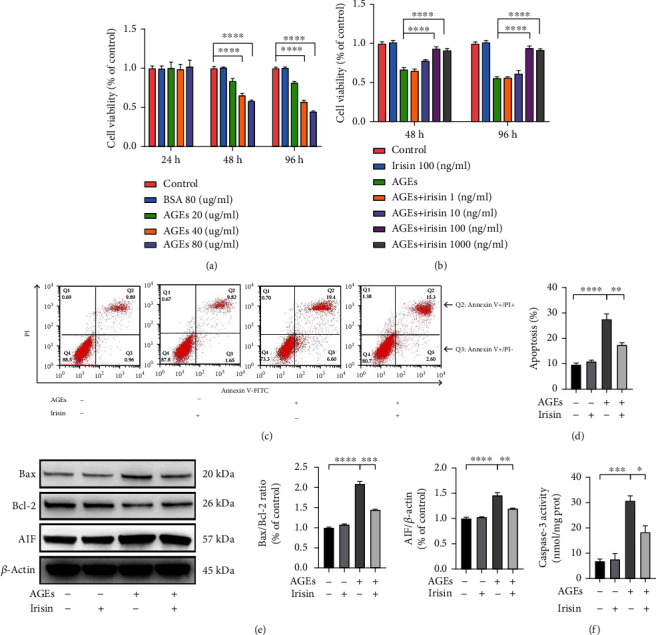
Irisin mitigated AGE-induced cell injury in ASCs. (a, b) Cells were treated with AGEs at different concentrations for 24, 48, or 96 h (a) and incubated with various concentrations of irisin along with 40 *μ*g/ml AGEs for 48 or 96 h (b). Cell viability was estimated using CCK-8 analysis. (c, d) Representative images of flow cytometric analysis by Annexin V-FITC/PI dual staining. Apoptotic cells are represented by the percentage of Annexin-V single-positive plus Annexin-V/PI double-positive cells. (e) Representative images of the WB assay and quantification of the Bax/Bcl-2 ratio and the expression level of AIF normalized to the expression levels of the housekeeping protein *β*-actin. (f) Caspase-3 activities in each group were detected. All results are representative of three independent experiments, and values are presented as mean ± SEM. ^∗^*P* < 0.05, ^∗∗^*P* < 0.01, ^∗∗∗^*P* < 0.001, and ^∗∗∗∗^*P* < 0.0001.

Isolated ASCs displayed osteogenic and adipogenic potential after culturing in the respective induction media, and they were positive for CD29, CD44, and CD73 (mesenchymal stromal cell markers) and negative for CD34 and CD45 (hematopoietic cell markers) (Figure S1). As shown by the CCK-8 assay results, AGEs hindered the proliferation of ASCs in a dose- and time-dependent manner (Figure 1(a)). Following treatment with AGEs at a low concentration of 20 *μ*g/ml, ASCs maintained high proliferative capacities for 24, 48, and 96 h. However, cell viability was reduced significantly after treatment with 40 *μ*g/ml or 80 *μ*g/ml AGEs for 48 and 96 h. Moreover, the control BSA at 80 *μ*g/ml did not display a significant impact on cell viability. We also evaluated the effects of different concentrations of irisin (0, 10, 100, and 1000 ng/ml) on the proliferation of ASCs exposed to 40 *μ*g/ml AGEs, and we observed that the reduced cell viability induced by AGEs could be significantly rescued by irisin in a dose-dependent manner, with concentrations from 1 ng/ml to 100 ng/ml (Figure 1(b)). However, the protective effects of irisin on cell viability were not significantly different between 100 ng/ml and 1000 ng/ml. Thus, we used 100 ng/ml irisin in subsequent experiments.

To further assess the impacts of AGEs and irisin on the apoptosis of ASCs, we used Annexin V and PI staining and flow cytometry. Apoptosis induced by AGEs was detected after 40 *μ*g/ml treatment for 24 h, and this proapoptotic effect could be hindered by treatment with 100 ng/ml irisin (Figures 1(c) and 1(d)), with no significant differences in apoptosis observed in ASCs treated with 100 ng/ml irisin alone. As members of the Bcl-2 family play important roles in initiating the mitochondrial apoptosis cascade, Bcl-2 and Bax expressions were detected by immunoblotting. Compared with the control group, 40 *μ*g/ml AGEs treatment induced proapoptotic Bax expression, while additional treatment with 100 ng/ml irisin reversed the elevated levels of Bax (Figure 1(e)). However, Bcl-2 levels decreased notably as a result of 40 *μ*g/ml AGEs treatment, and this effect could be alleviated by the treatment of 100 ng/ml irisin. The Bax/Bcl-2 ratio in ASCs increased remarkably after AGEs stimulation, while irisin treatment reversed AGE-induced changes in this ratio. Moreover, the expression of AIF and the activity of caspase-3 were also examined. As shown in Figures 1(e) and 1(f), 40 *μ*g/ml AGEs induced increases in AIF expression and caspase-3 activity, but these changes could be mitigated by treatment with 100 ng/ml irisin. The above results revealed that irisin treatment greatly attenuated AGE-induced cell injuries in ASCs.

### 3.2. Irisin Improves the Osteogenic Differentiation Capacity of ASCs Exposed to AGEs In Vitro

**Figure 2:**
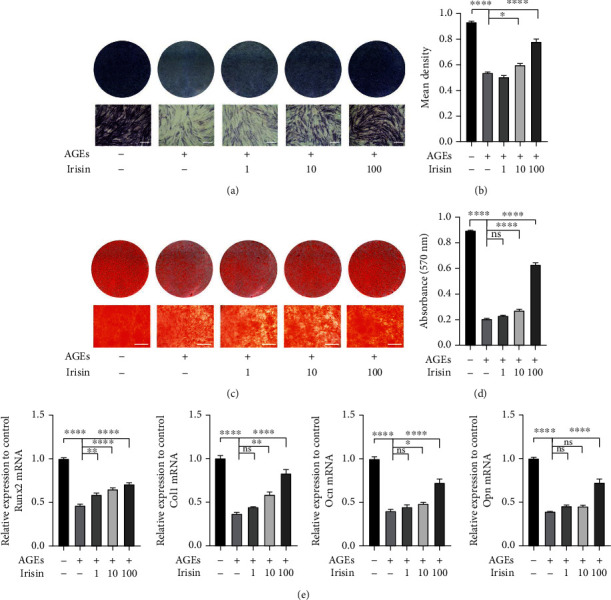
Irisin improved the osteogenic differentiation of ASCs exposed to AGEs in vitro. (a–d) Osteogenic differentiation was assessed by ALP staining on day 7 (a) and semiquantitative analysis of ALP staining (b) and ARS on day 21 (c). Calcium deposition was assessed by measuring the optical density at 570 nm (d). (e) qPCR analysis of the expression of the osteogenesis-specific genes *Runx2*, *Col1*, *Ocn*, and *Opn* (*β*-actin was used as the internal control). All results are representative of three independent experiments, and values are presented as mean ± SEM. Scale bar = 400 *μ*m. ALP: alkaline phosphatase; ARS: Alizarin red staining. ^∗^*P* < 0.05, ^∗∗^*P* < 0.01, ^∗∗∗^*P* < 0.001, and ^∗∗∗∗^*P* < 0.0001.

To evaluate the effects of AGEs and irisin on the osteogenesis of ASCs, we performed ALP staining and ARS on day 7 and day 21 after osteogenic induction, respectively. In comparison with the nontreated control group, the 40 *μ*g/ml AGE-treated group showed decreased ALP levels and mineralized nodule formation (Figures 2(a)–2(d)). However, the reduced osteogenic differentiation potential of ASCs stimulated with 40 *μ*g/ml AGEs was partially alleviated by additional treatment with irisin in a dose-dependent manner, with obviously reversed effects at a concentration of 100 ng/ml irisin.

Furthermore, the expression of the osteogenic-specific genes *Runx2*, *Col1*, *Opn*, and *Ocn* was measured by qPCR. The expression levels of the above genes were significantly lowered in the 40 *μ*g/ml AGE-treated group than in the control group, and irisin attenuated these adverse effects in a dose-dependent manner at day 7 after osteogenic induction (Figure 2(e)). Collectively, these findings demonstrated that AGEs inhibit ASC osteogenic differentiation potential, while irisin treatment alleviates this inhibitory effect.

### 3.3. Irisin Alleviates AGE-Induced Suppression of Cell Viability and Osteogenic Differentiation by Increasing SIRT3 In Vitro

**Figure 3:**
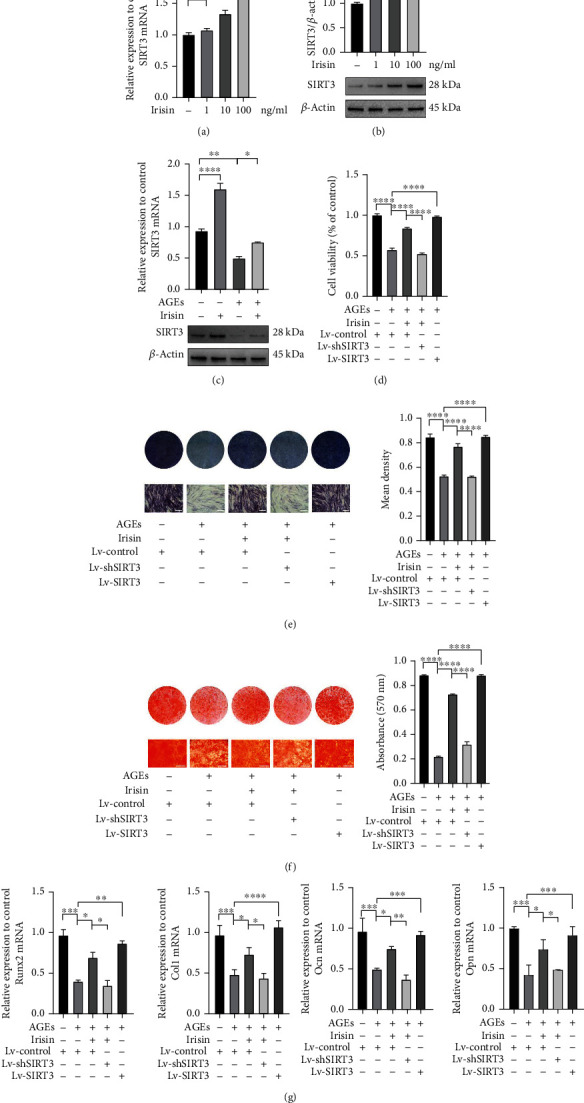
Irisin alleviated AGE-induced suppression of cell viability and osteogenic differentiation by increasing SIRT3 expression in vitro. (a, b) The gene and protein expression levels of SIRT3 were assessed by qPCR (a) and WB analysis (b) with different concentrations of irisin treatment. (c) The gene and protein expression levels of SIRT3 in ASCs treated with or without irisin under AGE-induced conditions. (d) Cells were transfected with lentiviruses that either overexpress or silence SIRT3, and cell viability was evaluated by CCK-8 assay. (e, f) Osteogenic differentiation was determined by ALP staining on day 7 and semiquantitative analysis of ALP staining (e) and ARS on day 21. Calcium deposition was assessed by measuring the optical density at 570 nm (f). (g) qPCR analysis of the expression of the osteogenic-specific genes *Runx2*, *Col1*, *Ocn*, and *Opn* (*β*-actin was used as the internal control). All results are representative of three independent experiments, and values are presented as mean ± SEM. Scale bar = 400 *μ*m. Lv-Control: control lentivirus; Lv-SIRT3: lentivirus-mediated SIRT3 overexpression; Lv-shSIRT3: lentivirus-mediated SIRT3 knockdown. ^∗^*P* < 0.05, ^∗∗^*P* < 0.01, ^∗∗∗^*P* < 0.001, and ^∗∗∗∗^*P* < 0.0001.

We next investigated the mechanism through which irisin modulated the cell viability and osteogenic differentiation capacities of ASCs in an AGE-stimulated environment. We found that after treatment with various concentrations of irisin for 48 h, both gene and protein expressions of SIRT3 within ASCs increased in a dose-dependent manner, with most obviously elevated SIRT3 expressions when exposed to 100 ng/ml irisin treatment (Figures 3(a) and 3(b)). And gene and protein expression of SIRT3 was significantly decreased with 40 *μ*g/ml AGEs incubation for 48 h compared to the nontreated control group; however, the suppressed expression of SIRT3 was greatly increased after treatment with 100 ng/ml irisin (Figure 3(c)). We then performed lentivirus-mediated SIRT3 overexpression or knockdown experiments in ASCs to further address how SIRT3 mediated the influences of irisin on the cell proliferation and osteogenic differentiation of ASCs. The overexpression and knockdown efficiencies were confirmed by qPCR and WB analysis (Figure S2).

Next, we examined the effects of SIRT3 overexpression and knockdown on the irisin-mediated effects on ASCs under AGEs exposure. CCK-8 assay results showed that the restored cell viability of ASCs induced by 100 ng/ml irisin treatment could be abolished by transfection with Lv-shSIRT3 (Figure 3(d)). In addition, knockdown of SIRT3 inhibited the irisin-mediated promotion of ASC osteogenic differentiation following 40 *μ*g/ml AGE treatment, as demonstrated by ALP staining and ARS (Figures 3(e) and 3(f)). We also assessed the mRNA levels of the osteogenic-specific genes *Runx2*, *Col1*, *Opn*, and *Ocn*. The results of qPCR analysis (Figure 3(g)) were consistent with the results of ALP staining and ARS, demonstrating that SIRT3 knockdown suppressed the ability of irisin treatment to rescue the osteogenic differentiation potential of ASCs treated with AGEs. However, the AGE-induced inhibition of cell proliferation and osteogenesis could be partially reversed by SIRT3 overexpression in ASCs, as indicated by the results of gene expression and mineralized nodule formation assessments (Figures 3(c)–3(g)).

### 3.4. Irisin Ameliorates AGE-Induced Mitochondrial Dysfunctions in ASCs via the SIRT3-Dependent Pathway

**Figure 4:**
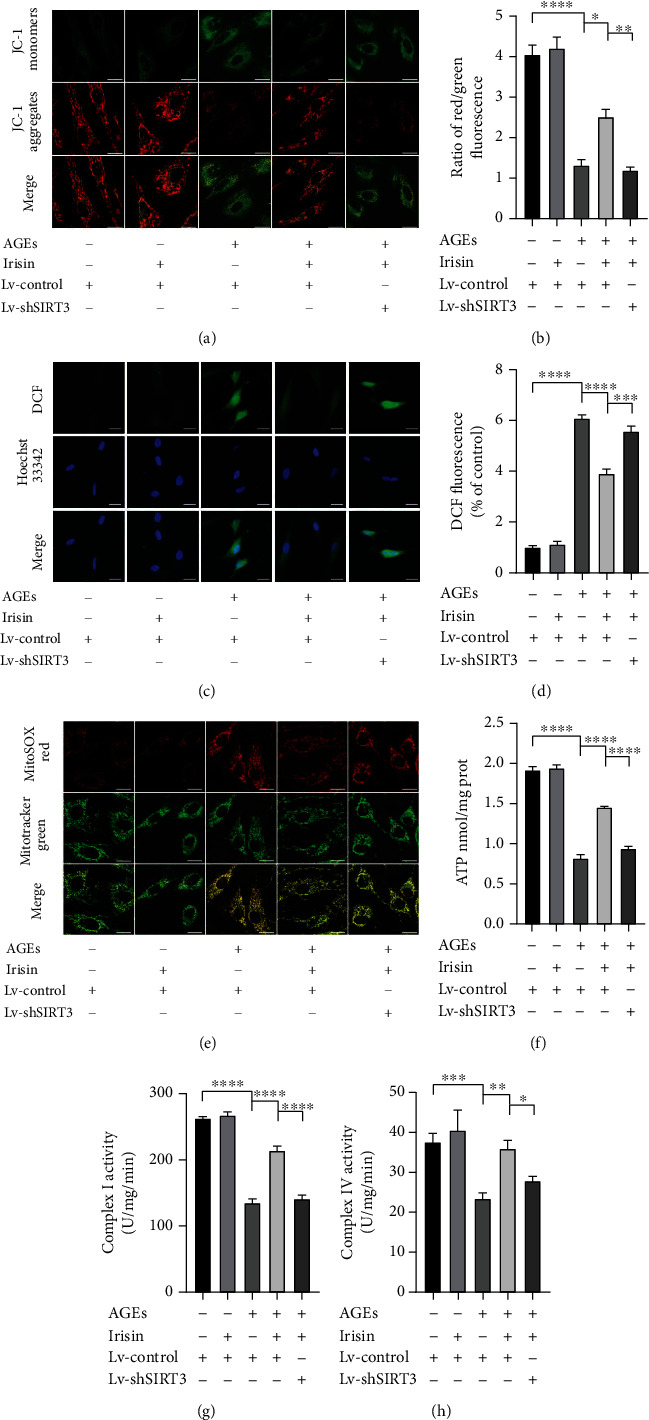
Irisin reversed AGE-induced mitochondrial dysfunction in ASCs via a SIRT3-dependent pathway. (a) *Δψ*m was assessed by using CLSM. Red fluorescence was emitted by JC-1 aggregates in healthy mitochondria with polarized inner mitochondrial membranes, whereas green fluorescence was emitted by cytosolic JC-1 monomers, indicating *Δψ*m depolarization. Merged images indicate the colocalization of JC-1 aggregates and monomers. (b) *Δψ*m of each group was calculated as the ratio of red to green fluorescence. (c) The intracellular ROS levels were estimated using DCF (green), and the fluorescence values were read at an excitation wavelength of 488 nm and emission wavelength of 525 nm. (d) The bar charts show the quantification of intracellular ROS levels expressed as the fold change relative to the control group. (e) The mitochondrial O_2_^·−^ levels were estimated using MitoSOX Red (red), and mitochondria were stained with MitoTracker Green Dye (green). (f–h) ATP content (f). The activity levels of mitochondrial electron transport chain complex I (g) and complex IV (h) were determined using corresponding assay kits according to the manufacturer's instructions. All results are representative of three independent experiments, and values are presented as mean ± SEM. Scale bar = 20 *μ*m. *Δψ*m: mitochondrial membrane potential; CLSM: confocal laser scanning microscopy; Lv-Control: control lentivirus; Lv-shSIRT3: lentivirus-mediated SIRT3 knockdown. ^∗^*P* < 0.05, ^∗∗^*P* < 0.01, ^∗∗∗^*P* < 0.001, and ^∗∗∗∗^*P* < 0.0001.

To elucidate the role of SIRT3 in mediating the effects of irisin on regulating the cell viability and osteogenic differentiation of ASCs with AGEs treatment, we carried out multiple assays to assess mitochondrial status and the functions involved in this process. We used a JC-1 probe to measure MMP (*Δψ*m) in ASCs. When exposed to 40 *μ*g/ml AGEs treatment, the cells displayed increased green fluorescence, suggesting depolarization of the mitochondrial membrane. However, 100 ng/ml irisin treatment mitigated the alterations in *Δψ*m in the AGE-stimulated group, as shown by weakened green fluorescence and strengthened red fluorescence. In addition, Lv-shSIRT3 transfection abolished the influences of irisin on AGE-induced alterations in *Δψ*m in ASCs (Figures 4(a) and 4(b). These results suggest that irisin significantly ameliorated AGE-induced depolarization of mitochondrial membrane mediated by SIRT3 in ASCs.

Next, we evaluated the effects of irisin on intracellular and mitochondrial redox status by using DCF and MitoSOX Red staining, respectively. As shown in Figures 4(c)–4(e), compared with the control condition, 40 *μ*g/ml AGEs treatment obviously increased intracellular ROS and mitochondrial O_2_^·−^ generation, but the enhanced accumulation of intracellular ROS and mitochondrial O_2_^·−^ was significantly lowered by 100 ng/ml irisin treatment. In addition, the effects of irisin were clearly abolished by Lv-shSIRT3 transfection.

Furthermore, we examined ATP synthesis status and mitochondrial respiratory chain complex activities to elucidate the effects of irisin on mitochondrial respiratory functions in ASCs. Compared with the control condition, 40 *μ*g/ml AGEs treatment greatly inhibited ATP generation and complex I and IV activities within ASCs, whereas these inhibitory effects of AGEs on mitochondrial respiratory functions could be obviously ameliorated by treatment with 100 ng/ml irisin (Figures 4(f)–4(h)). However, the knockdown of SIRT3 by lentivirus-mediated transfection partially suppressed the mitigating effects of 100 ng/ml irisin on ATP content and complex I and IV activities within ASCs. The above results suggest that irisin ameliorated AGE-induced mitochondrial dysfunction in ASCs in a SIRT3-dependent manner.

### 3.5. Irisin Reduces ROS Generation by Modulating SIRT3-Mediated SOD2 Deacetylation, Antioxidative Enzyme Activities, and Mitochondrial Biogenesis

**Figure 5:**
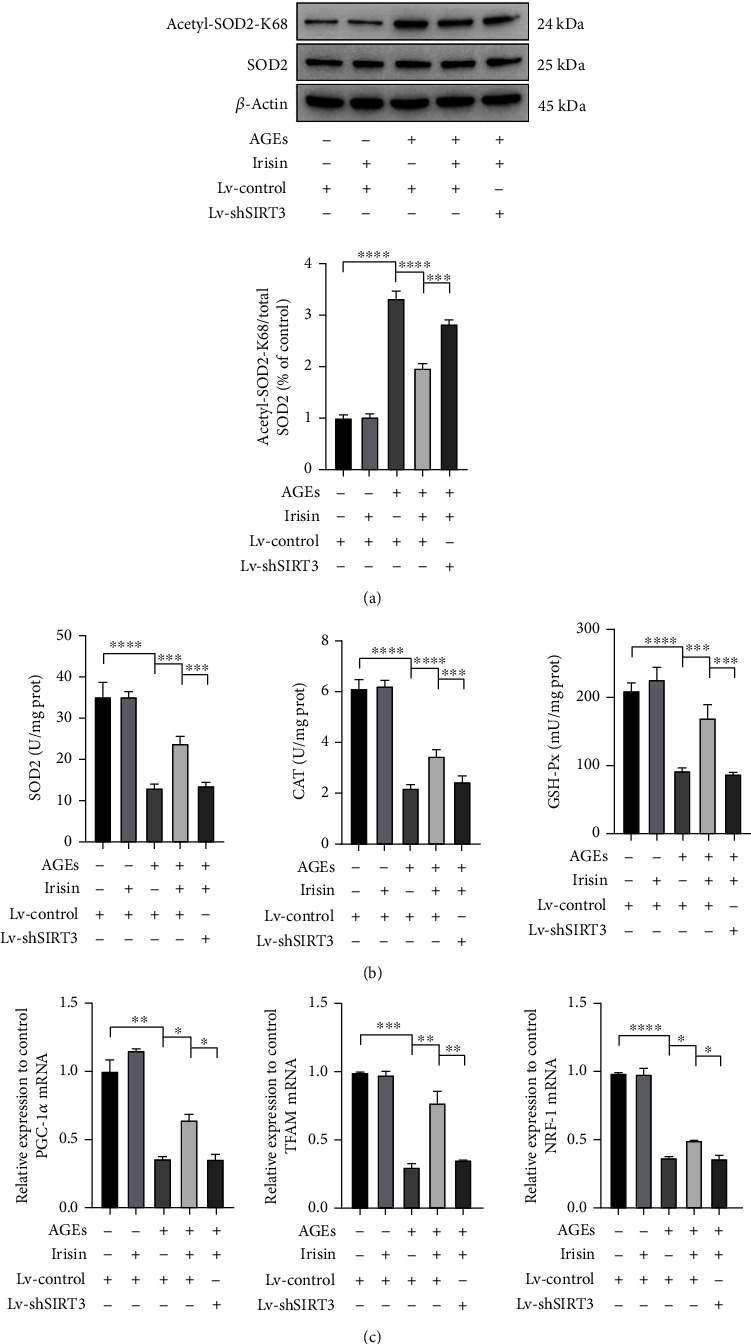
Irisin reduced ROS generation by stimulating SIRT3-mediated SOD2 deacetylation, mitochondrial enzyme activities, and mitochondrial biogenesis. (a) After indicated treatments, cells were harvested and lysed to detect the protein levels of acetyl-SOD2-K68 by WB analysis. The relative expressions of acetyl-SOD2-K68 were normalized by total SOD2. (b) The activities of the enzymes SOD2, CAT, and GSH-Px were determined using the corresponding assay kits. (c) The expression levels of mitochondrial biogenesis-related genes (PGC-1*α*, TFAM, and NRF-1) were normalized to the expression level of the housekeeping gene *β*-actin as measured by qPCR assay. All results are representative of three independent experiments, and values are presented as mean ± SEM. SOD2: superoxide dismutase 2; CAT: catalase; GSH-Px: glutathione peroxidase; Lv-Control: control lentivirus; Lv-shSIRT3: lentivirus-mediated SIRT3 knockdown. ^∗^*P* < 0.05, ^∗∗^*P* < 0.01, ^∗∗∗^*P* < 0.001, and ^∗∗∗∗^*P* < 0.0001.

As the intracellular ROS levels indicated by DCF were markedly elevated after AGEs treatment but additional treatment of irisin suppressed these changes in ASCs, we further explored the underlying mechanism. SOD2 is an important enzyme in mitochondria that eradicates ROS, and its activity is highly associated with its deacetylation status. According to previous studies, SOD2 is usually acetylated at K68 and K122 under stress conditions [29], which leads to a decrease in its ability to scavenge excessive ROS within cells. WB analysis showed that 40 *μ*g/ml AGEs treatment led to increased levels of K68 acetylation on SOD2, but the enhanced acetylation of SOD2 at K68 was greatly decreased with additional treatment of 100 ng/ml irisin. The above changes were partially reversed by SIRT3 knockdown (Figure 5(a)). Furthermore, we measured the activities of several ROS scavenging enzymes, such as SOD2, CAT, and GSH-Px. As shown in Figure 5(b), 40 *μ*g/ml AGEs treatment significantly disturbed the normal enzymatic activities of SOD2, CAT, and GSH-Px, whereas 100 ng/ml irisin incubation obviously mitigated these inhibitory effects. However, knockdown of SIRT3 clearly abolished the altered effects induced by irisin (Figure 5(b)), suggesting that SIRT3 contributed greatly to the irisin treatment-mediated clearance of excessive ROS in ASCs exposed to AGEs.

The intracellular levels of ROS not only depend upon radical scavenging by mitochondrial antioxidative enzymes but also correlate with the generation of ROS, byproducts of the oxidative phosphorylation process of the mitochondrial electron transport chain. Thus, we further assessed the expression of several genes related to mitochondrial biogenesis, including PGC-1*α*, TFAM, and NRF-1 (Figure 5(c)). The results showed that 40 *μ*g/ml AGEs treatment led to the downregulation of the expression of the above genes, whereas additional 100 ng/ml irisin treatment notably ameliorated the AGE-induced decrease in mitochondrial biogenesis gene transcription. Furthermore, knockdown of SIRT3 interfered with the irisin-mediated upregulation of the expression levels of PGC-1*α*, TFAM, and NRF-1. These results clearly indicate that irisin administration enhanced SIRT3 expression in ASCs, which was required not only for maintaining the scavenging capacities of antioxidative enzymes but also for activating mitochondrial biogenesis and decreasing the generation of ROS as a byproduct of oxidative phosphorylation in an AGE-induced environment.

### 3.6. Irisin Mitigates AGE-Induced Abnormal Mitophagy in ASCs in a SIRT3-Dependent Manner

**Figure 6:**
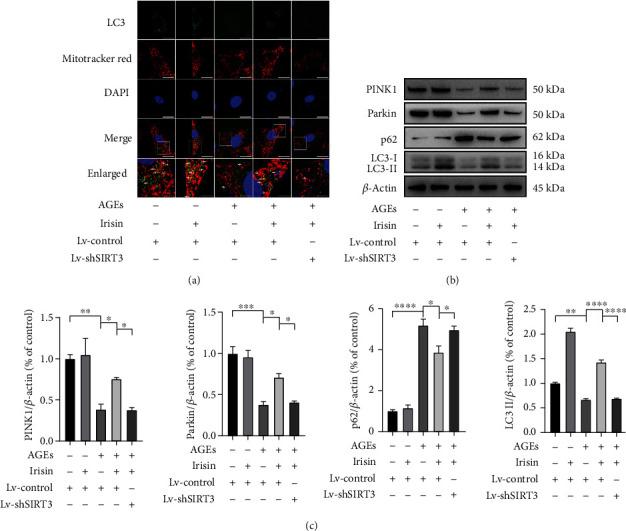
Irisin mitigates AGE-induced abnormal mitophagy in ASCs in a SIRT3-dependent manner. (a) Representative fluorescence images of triple staining with LC3 immunofluorescence (green), MitoTracker Red (red), and DAPI (blue) in ASCs with the indicated treatments (scale bar = 30 *μ*m in upper four panels, scale bar = 10 *μ*m in lowest panel). The lowest panel shows magnifications of the insets in each group, and white arrows, pointing to the merged yellow dots, indicate the colocalization of LC3 and MitoTracker Red staining. (b, c) Representative WB assay (b) and quantification (c) of relative expression levels of PINK1, Parkin, p62, and LC3-II proteins normalized to *β*-actin. All results are representative of three independent experiments, and values are presented as mean ± SEM. Lv-Control: control lentivirus; Lv-shSIRT3: lentivirus-mediated SIRT3 knockdown. ^∗^*P* < 0.05, ^∗∗^*P* < 0.01, ^∗∗∗^*P* < 0.001, and ^∗∗∗∗^*P* < 0.0001.

Since dysfunctional or damaged mitochondria are removed by a mechanism called mitophagy to maintain cellular homeostasis, we used LC3 immunofluorescence, MitoTracker Red and DAPI triple staining [30] to detect the effect of irisin and AGEs on mitophagy in ASCs. As shown in Figure 6(a), normal levels of mitophagy could be indicated by colocalization of LC3 and MitoTracker Red. After incubation with 40 *μ*g/ml AGEs for 48 h, LC3 expression within cells decreased significantly, while additional 100 ng/ml irisin treatment partially elevated LC3 fluorescence and increased the number of LC3-positive autophagosomes colocalized with MitoTracker Red staining. However, the mitigating effects of irisin on mitophagy could be hindered by Lv-shSIRT3 transfection.

Next, we investigated the protein expression levels of PINK1, Parkin, p62, and LC3-II, which are autophagy- or mitophagy-related proteins. As shown in Figures 6(b) and 6(c), the expression levels of PINK1, Parkin, and LC3-II decreased greatly, while the expression level of the autophagic substrate p62 increased with 40 *μ*g/ml AGEs incubation. In ASCs exposed to AGEs, additional treatment with 100 ng/ml irisin elevated PINK1, Parkin, and LC3-II expression and significantly decreased the autophagic substrate p62, while Lv-shSIRT3 transfection inhibited the above changes in autophagy- and mitophagy-related protein expressions stimulated by irisin. Taken together, these results showed that irisin ameliorated AGE-induced abnormal mitophagy in ASCs in a SIRT3-dependent manner.

### 3.7. Irisin Activates SIRT3 via the AMPK-PGC-1*α* Pathway in ASCs

**Figure 7:**
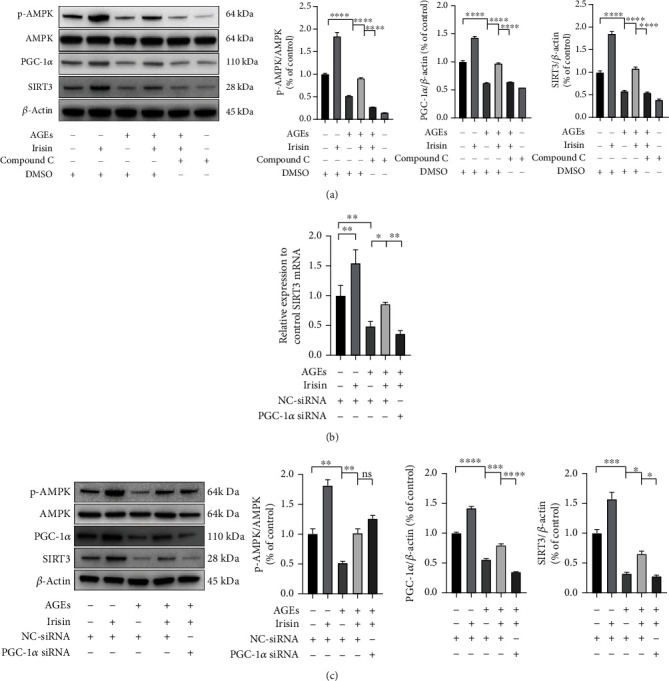
Irisin stimulated the AMPK-PGC-1*α*-SIRT3 signaling pathway in ASCs. (a) Representative images of p-AMPK, AMPK PGC-1*α*, and SIRT3 in each group after the indicated treatment, as determined by WB analysis. (b) The gene expression of the SIRT3 was determined by qPCR assay. (c) Effects of PGC-1*α* siRNA transfection on the AMPK/PGC-1*α*/SIRT3 signaling pathway in irisin-treated ASCs under AGE stimulation, as determined by WB analysis. All results are representative of three independent experiments, and values are presented as mean ± SEM. Lv-Control: control lentivirus; Lv-shSIRT3: lentivirus-mediated SIRT3 knockdown; NC-siRNA: negative control-siRNA. ^∗^*P* < 0.05, ^∗∗^*P* < 0.01, ^∗∗∗^*P* < 0.001, and ^∗∗∗∗^*P* < 0.0001.

As indicated in Figure 7(a), compared with the control condition, the administration of 100 ng/ml irisin increased the protein expression levels of p-AMPK, PGC-1*α*, and SIRT3. To explore the role of AMPK in this process, the strong AMPK inhibitor Compound C was added at a concentration of 10 *μ*M. Irisin-induced AMPK activation was hindered by Compound C pretreatment, and PGC-1*α* and SIRT3 expression was downregulated. To further address the regulatory role of PGC-1*α*, siRNA transfection was employed to transiently silence PGC-1*α*, and the transfection efficiencies were confirmed by qPCR and WB analysis (Figure S2). The downregulation of PGC-1*α* expression by siRNA transfection inhibited the expression of SIRT3 mRNA (Figure 7(b)) and protein (Figure 7(c)) in ASCs, implying that PGC-1*α* played a vital role in mediating the activation of SIRT3 expression by irisin. PGC-1*α* interference did not have obvious impacts on the expression of p-AMPK and AMPK. Our findings suggested that the AMPK-PGC-1*α*-SIRT3 signaling pathway was involved in mediating the effects of irisin on preserving cell viability and osteogenic differentiation potential in an AGE-induced environment.

### 3.8. Gel Encapsulated with ASCs and Irisin Promoted Bone Healing of Calvarial CSDs in T2DM Rat

**Figure 8:**
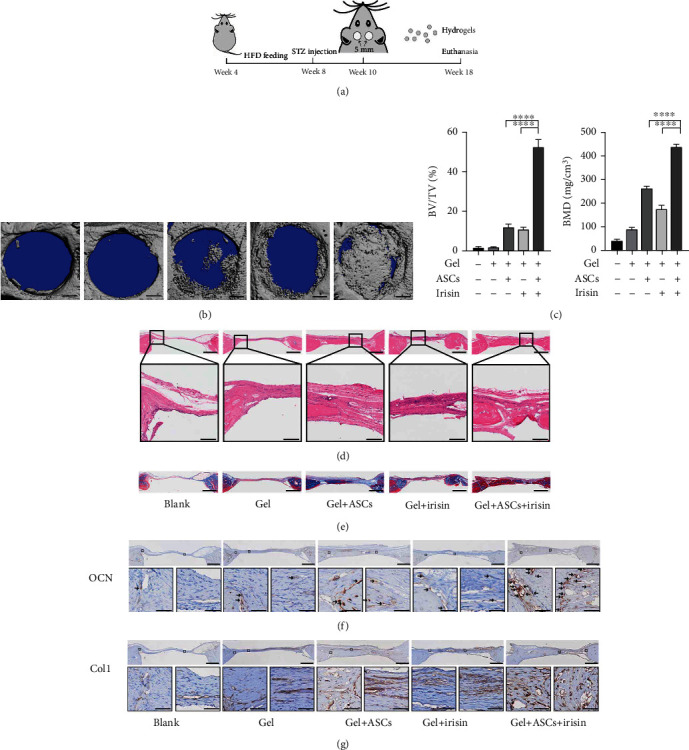
Transplantation of gels encapsulating ASCs and irisin promoted bone healing in CSDs in diabetic rats. (a) Timeline for the operation process in SD rats. (b) Representative micro-CT reconstruction images of rat CSDs (scale bar = 1 mm). (c) The microstructural parameters of the ROI, including BV/TV and BMD (*n* = 6). (d) Representative images of calvarial defect sections stained with HE (scale bar = 1 mm in upper images; scale bar = 200 *μ*m in lower images). The lower panels show magnifications of the insets in each group. (e) Representative images of calvarial defect sections stained with Masson's trichrome staining (scale bar = 1 mm) (the blue color indicates the regenerated bone, collagen fibers, or osteoid, while the red color indicates the mature bone). (f) Representative images of calvarial defect sections with immunohistochemical staining of OCN. The dark brown color represents the positive staining for OCN protein, with black arrows indicating OCN-positive staining. (g) Representative images of calvarial defect sections with immunohistochemical staining of Col1. The dark brown color represents the positive staining for Col1 protein. Scale bar = 1 mm in upper images, scale bar = 50 *μ*m in lower images in (f, g), and the lower panels show magnifications of the insets in each group. BV/TV: bone volume/total volume; BMD: bone mineral density. ^∗^*P* < 0.05, ^∗∗^*P* < 0.01, ^∗∗∗^*P* < 0.001, and ^∗∗∗∗^*P* < 0.0001.

We further assessed the effects of irisin on ASC osteogenesis in vivo. After establishing T2DM rat models, we created calvarial CSDs and transplanted gels encapsulating ASCs to investigate their effects on osteogenesis, as displayed in Figure 8(a). Representative three-dimensional reconstruction images based on micro-CT are shown in Figure 8(b). As indicated in the images, in the gels mixed with both ASCs and irisin, a significantly larger amount of newly formed bone appeared within defective areas than that in the group transplanted with gels with only ASCs, suggesting that irisin is involved in promoting the osteogenesis of ASCs in T2DM models. However, in groups with gels that did not contain ASCs, only a small amount of new bone formed around the border of calvarial CSDs even when gels were mixed with irisin. The microstructural parameters, including BV/TV and BMD, were also significantly higher in the group transplanted with irisin-pretreated ASCs and irisin than in other groups, as shown in Figure 8(c).

Histologic structures of CSDs areas were observed and analyzed with HE and MTC staining by using light microscopy (Figures 8(d) and 8(e)). In both the blank control and gel-only groups, a minimal amount of new bone formation was observed along the margin of the defects, with areas mainly occupied by connective tissues. In the group transplanted with ASCs alone, few newly formed bones appeared not only in the margin but also in the center of the defects, whereas only a few new bones formed around the margin of the defects with administration of irisin alone. However, in the group transplanted with both ASCs and irisin, a significant amount of thick newly formed bone was observed, with osseous islands fused and continuously bridged, almost covering the bone defect area.

In the MTC images, more collagen formation was observed in the group transplanted with both ASCs and irisin than in the other groups (Figure 8(e)). Regarding bone formation, the HE and MTC images acquired from different groups displayed trends consistent with those shown by the micro-CT analysis.

As late specific and nonspecific markers of osteoblastic differentiation and mineralization, OCN and Col1 were assessed by immunochemistry staining in paraffin sections of the calvarial defects. Positive staining of OCN as dark brown was dramatically increased in the group transplanted with irisin-pretreated ASCs and irisin compared to the other groups. Similarly, images of areas next to the defects showed that Col1 was prevalent in groups transplanted with ASCs or with irisin, while the staining had significantly greater intensity in the group transplanted with irisin-pretreated ASCs and irisin than in other groups (Figures 8(f) and 8(g)). The results showed that irisin-pretreated ASCs along with irisin greatly promoted new bone formation in the calvarial CSDs of diabetic rat models.

## 4. Discussion

In this study, we demonstrated that irisin alleviated the AGE-induced suppression of cell viability and osteogenic differentiation potential of ASCs. On the one hand, irisin regulated oxidative stress by modulating SIRT3-mediated mitochondrial respiratory functions, SOD2 acetylation status, and enzyme activities in ASCs under exposure to AGEs. On the other hand, irisin ameliorated the adverse effects of AGEs on mitochondrial biogenesis and mitophagy in a SIRT3-dependent manner. In addition, irisin pretreatment increased the osteogenesis of ASCs in calvarial CSDs in diabetic rats. Our findings highlight the role of irisin in promoting bone healing and indicate that irisin could be a novel therapeutic strategy in bone defect regeneration under diabetic conditions.

Increased formation and deposition of AGEs occur in tissues under chronic hyperglycemia, and this is accompanied by notably increased oxidative stress status [31, 32]. A previous study illustrated the increased accumulation of AGEs in the bone and adipose tissues of a diabetic osteoporosis mouse model [9]. In our in vitro cell models, we utilized AGEs to mimic the hyperglycemic, chronic inflammatory environment and found that AGEs hindered the cell growth of ASCs in a time- and dose-dependent manner and inhibited the osteogenic differentiation of ASCs.

Irisin, a myokine mainly released from skeletal muscles following exercise, is considered to impact multiple tissues, such as the cardiovascular system, adipose tissue, and bone, and to mediate the beneficial effects of exercise [33]. A variety of studies have shown that irisin can protect endothelial cells from apoptosis dysfunctions induced by AGEs [34]. Irisin can also mitigate chondrocyte dysfunction and osteoarthritis development by modulating autophagy [35, 36]. In addition, accumulating evidence has revealed the involvement of irisin in bone homeostasis and remodeling. Recent reports illustrated that irisin could help maintain osteoblast activity and promote osteoblastogenesis by downregulating cell senescence or stimulating autophagy [17, 37]. Moreover, irisin could promote the osteogenesis of MC3T3-E1 osteoblasts via AMPK-mediated macrophage polarization [38]. In the current study, we observed that moderate concentrations of irisin (100 ng/ml) clearly mitigated the suppression of cell viability and reversed the inhibition of osteogenesis induced by AGEs in ASCs.

Of note, we also found that SIRT3 expression within ASCs was downregulated by 40 *μ*g/ml AGEs treatment, whereas irisin treatment restored SIRT3 expression, as partially illustrated by WB analysis. Multiple studies have documented that SIRT3 plays a regulatory role in bone metabolism and homeostasis. During the osteogenesis of the preosteoblastic cell line MC3T3-E1, the expression of SIRT3 was markedly upregulated [39] while the osteogenic potential of this cell line was hindered by the depletion of the SIRT3 gene [23], suggesting that SIRT3 is essential for the osteogenic differentiation of preosteoblasts. Moreover, knockdown of SIRT3 led to increased osteoclast activities and bone resorption, which resulted in significant bone mass loss [40]. In line with other studies [41], we demonstrated that SIRT3 was essential for the osteogenic differentiation of ASCs under AGE-induced conditions, as indicated by the decreases in mineralized nodule formation and osteogenic-related gene expression induced by SIRT3 knockdown. However, the inhibited osteogenesis could be reversed by SIRT3 overexpression under similar treatment conditions.

A variety of studies have suggested that the oxidative environment within osteoporosis, diabetes, or other metabolic diseases facilitates MSC defects through the induction of mitochondrial dysfunction and excessive ROS accumulation [42]. We examined the mitochondrial functions of ASCs and found that the MMP depolarized significantly with AGEs treatment, while additional irisin treatment reversed this change, suggesting that irisin helped to preserve the mitochondrial status to maintain the physiological processes within ASCs. Then, we detected intracellular ROS and mitochondrial O_2_^·−^ levels, which revealed the accumulation of large amounts of ROS in cells after AGEs stimulation. However, through treatment with irisin, the intracellular ROS and mitochondrial O_2_^·−^ levels were obviously decreased, indicating that there might be an imbalance between ROS generation and scavenging. Our findings confirmed the protective impacts of irisin on AGE-induced cell injuries and excessive intracellular and mitochondrial oxidative stress, which is in line with one previous study concerning endothelial cells [34].

Given that increasing evidence indicates the vital role of SIRT3, an important deacetylase mainly within mitochondria, in modulating ATP synthesis, ROS generation, mitochondrial dynamics, and mitophagy [43, 44], we sought to further investigate whether SIRT3 exerts a protective effect by attenuating AGE-induced injuries within ASCs. To elucidate the role of SIRT3 in this process, we used a lentivirus-mediated shSIRT3 knockdown technique to further disclose the underlying mechanism. Our study revealed that SIRT3 knockdown enhanced intracellular ROS and mitochondrial O_2_^·−^ accumulation, thus deteriorating its proliferative and osteogenic differentiation capacities, indicating the role of SIRT3 in modulating oxidative stress to maintain bone homeostasis. According to previous studies, SIRT3 modulates intracellular oxidative stress by directly activating the antioxidant capacity of a variety of targets via deacetylation [29, 45]. Therefore, we further explored the underlying molecular mechanism by investigating the activities of enzymes involved in eradicating intracellular ROS, such as SOD2, CAT, and GSH-Px, which are regulated by the deacetylase SIRT3 [39, 46]. Accumulating studies have mentioned that SIRT3 can regulate SOD2 activities by altering the deacetylation status at K68 and K122 [29]. In the present study, the activities of the enzymes SOD2, CAT, and GSH-Px in ASCs were decreased significantly by AGEs treatment; however, irisin increased their activities in a SIRT3-dependent manner. We also detected the acetylation status of SOD2. Our findings indicated that irisin incubation promoted a decrease in the AGE-induced increased acetylation level of SOD2 at K68, but this effect was counteracted by Lv-shSIRT3 knockdown. These results were consistent with the findings from another study that focused on elucidating the modulatory role of SIRT3 in the deleterious effect of dexamethasone on bone marrow-derived stem cells (BMSCs) [41].

Mitophagy is a mechanism by which damaged mitochondria are selectively degraded by autophagy to maintain mitochondrial quality and function [47], which ensures mitochondrial homeostasis. However, mitophagy can also be affected under some pathological conditions, resulting in the disturbance of cellular physiological functions [48]. Previous evidence has revealed that abnormal mitophagy is closely linked to dysfunctions in bone marrow stem cells and other bone metabolism disorders [24, 49]. In our study, we found that AGEs treatment disturbed the mitophagy process in ASCs, which may hinder the clearance of dysfunctional or damaged mitochondria and account for the disruption in cellular homeostasis. Of note, irisin ameliorated AGE-induced abnormal mitophagy in ASCs in a SIRT3-dependent manner. These findings were consistent with previous studies, which also suggested that SIRT3-mediated mitophagy plays a vital role in initiating and progressing age-associated osteoporosis [24]. In our present study, mitophagy-related PINK1 and Parkin did not change significantly while autophagy-related LC3-II increased with irisin treatment in the absence of AGEs exposure. Macroautophagy/autophagy is an evolutionarily conserved degradation system in which intracellular contents such as proteins, organelles, and lipids are degraded in a lysosome dependent manner [50], while organelle-specific autophagy is an essential mechanism for cellular homeostasis by removing the specific substrates, namely, dysfunctional or redundant organelles [51, 52]. Until now, multiple types of organelle-specific autophagy have been reported with regard to different organelles, including mitophagy, pexophagy, reticulophagy, ribophagy, lysophagy, and nucleophagy [53]. One potential reason that accounts for the different protein expressions may be the irisin treatment alone did not affect mitochondrial functions greatly; thus, no obvious mitophagy was initiated, while irisin treatment may induce changes to other organelles or intracellular processes, which may lead to increased autophagy targeting other specific organelles or proteins rather than mitochondria, with the elevated expression of LC3-II. The specific potential mechanism still needs further research.

In contrast to mitophagy, which eradicates dysfunctional mitochondria, mitochondrial biogenesis primarily contributes to cellular and mitochondrial homeostasis through the division of preexisting mitochondria via an autoreplication mechanism that increases the number of new and functional mitochondria. Notably, we also identified that mitochondrial biogenesis may account for the reduction in intracellular ROS levels, at least to some extent, via the SIRT3-mediated pathway. The increase in mitochondrial biogenesis under irisin treatment helped to restore healthy mitochondria to maintain mitochondrial oxidative phosphorylation and prevent excessive ROS generation, a mechanism that was also suggested in other studies [54].

Furthermore, the favorable effects of irisin against the inhibitory effects of AGEs prompted us to explore the underlying signaling pathway. AMPK is regarded as a key regulator of cell metabolism that maintains cellular functions [45]. In accordance with previous studies, we found that irisin activated the AMPK/PGC-1*α*/SIRT3 signaling pathway to regulate mitochondrial homeostasis and reduce oxidative stress [55, 56]. Since we identified the role of SIRT3 in mediating the restoration of osteogenic differentiation potential by irisin and revealed the SIRT3/ROS mechanism to some extent, further investigations into the molecular mechanism concerning the downstream signaling or crosstalk with other signaling pathways mediated by SIRT3 during the above processes are also needed.

After establishing diabetic rat models, we created CSDs and transplanted gels encapsulating ASCs to investigate their effects on osteogenesis. The data from our study suggested that irisin has strong potential to promote the osteogenesis of ASCs under diabetic conditions. Regarding MSC transplant therapies, several studies illustrated that the underlying therapeutic mechanisms include engraftment [57], paracrine signaling [58], and other effects, such as exosomes [59]. Since we found that irisin could enhance cell viability under AGE induction conditions in vitro and that gels encapsulating irisin pretreated ASCs along with irisin promoted more newly formed bone than the gels encapsulating only irisin without cells, we speculated that the cell-autonomous effects exerted by the ASCs within the engraftment may contribute largely to increased osteogenesis in animal models in vivo. However, the detailed mechanism underlying this process needs to be further clarified.

Although our present study revealed that transplantation of the gels encapsulating irisin-pretreated ASCs along with irisin resulted in better bone healing in CSDs than other groups, there is still an issue regarding the effective concentration and duration of irisin in situ. Until now, very few researches have been found about the degradation of irisin and measured irisin levels in serum or tissues of human or other rodent species, due to difficulty in preparing the samples and lack of reliable and sensitive antibodies [60]. One study reported a short half-life (1 h) of injected recombinant irisin in mice [61]. In order to examine the regulation of bone remodeling by irisin in vivo, they injected recombinant irisin protein daily into mice for 6 days and found that these injections raised the sclerostin mRNA level in osteocyte-enriched bones as well as the protein level in plasma even though a half-life of recombinant irisin in vivo is less than an hour. In addition, they also showed that irisin was also incredibly potent as 10 pM irisin could trigger phosphorylation events. By encapsulating the hormone-like myokine in slow-release gels, we assumed that irisin could exert potential effects even at extremely low concentrations as time lasted. However, this issue mentioned above still needs to be addressed in the future.

## 5. Conclusions

In summary, we demonstrated that irisin ameliorated the AGE-induced changes in the cell viability and osteogenic differentiation potential of ASCs. Irisin not only decreased excessive intracellular oxidative stress by modulating SIRT3-mediated mitochondrial respiratory functions and enzyme activities under exposure to AGEs but also irisin regulates mitochondrial biogenesis and mitophagy in a SIRT3-dependent manner. Furthermore, transplant gels encapsulated with irisin-pretreated ASCs along with irisin largely promoted bone healing in calvarial CSDs in diabetic rats. Our findings indicated the role of irisin in osteogenesis and targeting SIRT3 as a novel therapeutic intervention strategy for bone regeneration under diabetic conditions.

## Figures and Tables

**Table 1:** Sequences of the PCR primers for amplification of expressed genes.

Genes	Forward	Reverse
Runx2	5′GCACCCAGCCCATAATAGA 3′	5′TTGGAGCAAGGAGAACCC 3′
Col1	5′CCTACAGCACGCTTGTGGAT 3′	5′ATTGGGATGGAGGGAGTTTA 3′
Ocn	5′GAGGGCAGTAAGGTGGTGAATAG 3′	5′CGTCCTGGAAGCCAATGTG 3′
Opn	5′TGGTTTGCCTTTGCCTGTTCG 3′	5′ATGGCTTTCATTGGAGTTGCTTG 3′
SIRT3	5′TGCACGGTCTGTCGAAGGTC 3′	5′TGTCAGGTTTCACAACGCCAG 3′
PGC-1*α*	5′AGCCTCTTTGCCCAGATCTT 3′	5′GCAATCCGTCTTCATCCACC 3′
TFAM	5′GATCATGACGAGTTCTGCCG 3′	5′AGAACTTCACAAACCCGCAC 3′
NRF-1	5′ACATACTCAACTCCACGGCA 3′	5′ATGTGGCTCTGAGTTTCCGA 3′
*β*-Actin	5′AATCGTGCGTGACATTAAAGAG 3′	5′CATTGCCGATAGTGATGACCT 3′

## Data Availability

The data used to support the findings of this study are available from the corresponding author upon request.

## Abbreviations

ASC: Adipose-derived stem cell
AGEs: Advanced glycation end products
BSA: Bovine serum albumin
SIRT3: Sirtuin 3
ROS: Reactive oxygen species
DCFH-DA: 2′,7′-Dichlorodihydrofluorescein diacetate
LC3: Microtubule associated protein 1 light chain 3
NAD^+^: Nicotinamide adenine dinucleotide
MMP: Mitochondrial membrane potential
qPCR: Quantitative polymerase chain reaction
CSD: Critical-sized defect
AMPK: AMP-activated protein kinase
AIF: Apoptosis-inducing factor
PINK1: PTEN-induced kinase 1
PGC-1*α*: Proliferator-activated receptor *γ* coactivator-1*α*
SOD2: Superoxide dismutase 2
CAT: Catalase
GSH-Px: Glutathione peroxidase
NRF-1: Nuclear transcription factor 1
TFAM: Mitochondrial transcription factor A.

## Supplementary Materials

Supplementary Materials Figure S1: Identification of ASCs in vitro. (a) Assessment of the osteogenic potential of ASCs via ARS on day 21 (scale bar = 400 *μ*m). (b) Assessment of adipogenic induction in ASCs via oil red O staining on day 14. (c) ASCs were positive for MSC markers (CD29, CD44, and CD73) but negative for hematopoietic markers (CD34 and CD45). S2: overexpression and knockdown transfection efficiencies for SIRT3 and PGC-1*α*. Overexpression transfection efficiencies by Lv-SIRT3 (a) and knockdown transfection efficiencies by Lv-shSIRT3 (b) and by PGC-1*α*-siRNA (c) were detected by qPCR (upper panel) and WB (lower panel). ^∗^*P* < 0.05, ^∗∗^*P* < 0.01, ^∗∗∗^*P* < 0.001, and ^∗∗∗∗^*P* < 0.0001.

## References

[1] Whiting D., Guariguata L., Weil C., Shaw J. (2011). IDF diabetes atlas: global estimates of the prevalence of diabetes for 2011 and 2030. *Diabetes Research and Clinical Practice*.

[2] Marin C., Luyten F., Van der Schueren B., Kerckhofs G., Vandamme K. (2018). The impact of type 2 diabetes on bone fracture healing. *Frontiers in Endocrinology*.

[3] Camargo W., de Vries R., van Luijk J. (2017). Diabetes mellitus and bone regeneration: a systematic review and meta-analysis of animal studies. *Tissue Engineering Part B: Reviews*.

[4] Grayson W., Bunnell B., Martin E., Frazier T., Hung B., Gimble J. (2015). Stromal cells and stem cells in clinical bone regeneration. *Nature Reviews. Endocrinology*.

[5] Gimble J., Ray S., Zanata F. (2017). Adipose derived cells and tissues for regenerative medicine. *ACS Biomaterials Science & Engineering*.

[6] Tan J., Zhou L., Zhou Y. (2017). The influence of diabetes mellitus on proliferation and osteoblastic differentiation of MSCs. *Current Stem Cell Research & Therapy*.

[7] Chaudhuri J., Bains Y., Guha S. (2018). The role of advanced glycation end products in aging and metabolic diseases: bridging association and causality. *Cell Metabolism*.

[8] Ferrari S., Eastell R., Napoli N. (2020). Denosumab in postmenopausal women with osteoporosis and diabetes: subgroup analysis of FREEDOM and FREEDOM extension. *Bone*.

[9] Volpe C. M. O., Villar-Delfino P. H., Dos Anjos P. M. F., Nogueira-Machado J. A. (2018). Cellular death, reactive oxygen species (ROS) and diabetic complications. *Cell Death & Disease*.

[10] Madsen-Bouterse S. A., Mohammad G., Kanwar M., Kowluru R. A. (2010). Role of mitochondrial DNA damage in the development of diabetic retinopathy, and the metabolic memory phenomenon associated with its progression. *Antioxidants & Redox Signaling*.

[11] Bock Florian J., Tait Stephen W. G. (2020). Mitochondria as multifaceted regulators of cell death. *Nature Reviews. Molecular Cell Biology*.

[12] Li Y., Wang L., Zhang M. (2020). Advanced glycation end products inhibit the osteogenic differentiation potential of adipose-derived stem cells by modulating Wnt/*β*-catenin signalling pathway via DNA methylation. *Cell Proliferation*.

[13] Boström P., Wu J., Jedrychowski M. (2012). A PGC1-*α*-dependent myokine that drives brown-fat-like development of white fat and thermogenesis. *Nature*.

[14] Perakakis N., Triantafyllou G., Fernández-Real J. (2017). Physiology and role of irisin in glucose homeostasis. *Nature Reviews. Endocrinology*.

[15] Cao R., Zheng H., Redfearn D., Yang J. (2019). FNDC5: a novel player in metabolism and metabolic syndrome. *Biochimie*.

[16] Park H., Kim H., Zhang D., Yeom H., Lim S. (2019). The novel myokine irisin: clinical implications and potential role as a biomarker for sarcopenia in postmenopausal women. *Endocrine*.

[17] Chen X., Sun K., Zhao S. (2020). Irisin promotes osteogenic differentiation of bone marrow mesenchymal stem cells by activating autophagy via the Wnt//*β*-catenin signal pathway. *Cytokine*.

[18] Chen Z., Zhang Y., Zhao F. (2020). Recombinant Irisin prevents the reduction of osteoblast differentiation induced by stimulated microgravity through increasing *β*-catenin expression. *International Journal of Molecular Sciences*.

[19] Chen T., Peng Y., Hu W. (2022). Irisin enhances chondrogenic differentiation of human mesenchymal stem cells via Rap1/PI3K/AKT axis. *Stem Cell Research & Therapy*.

[20] Finkel T., Deng C., Mostoslavsky R. (2009). Recent progress in the biology and physiology of sirtuins. *Nature*.

[21] Denu R. (2017). SIRT3 enhances mesenchymal stem cell longevity and differentiation. *Oxidative Medicine and Cellular Longevity*.

[22] Guan C., Huang X., Yue J. (2021). SIRT3-mediated deacetylation of NLRC4 promotes inflammasome activation. *Theranostics*.

[23] Ding Y., Yang H., Wang Y., Chen J., Ji Z., Sun H. (2017). Sirtuin 3 is required for osteogenic differentiation through maintenance of PGC-1ɑ-SOD2-mediated regulation of mitochondrial function. *International Journal of Biological Sciences*.

[24] Guo Y., Jia X., Cui Y. (2021). Sirt3-mediated mitophagy regulates AGEs-induced BMSCs senescence and senile osteoporosis. *Redox Biology*.

[25] Wei X., Li G., Yang X. (2013). Effects of bone morphogenetic protein-4 (BMP-4) on adipocyte differentiation from mouse adipose-derived stem cells. *Cell Proliferation*.

[26] Li G., Wang M., Hao L. (2014). Angiotensin II induces mitochondrial dysfunction and promotes apoptosis via JNK signalling pathway in primary mouse calvaria osteoblast. *Archives of Oral Biology*.

[27] Xu X., Fang K., Wang L., Liu X., Zhou Y., Song Y. (2019). Local application of semaphorin 3A combined with adipose-derived stem cell sheet and anorganic bovine bone granules enhances bone regeneration in type 2 diabetes mellitus rats. *Stem Cells International*.

[28] Wang H., Hu B., Li H. (2022). Biomimetic mineralized hydroxyapatite nanofiber-incorporated methacrylated gelatin hydrogel with improved mechanical and osteoinductive performances for bone regeneration. *International Journal of Nanomedicine*.

[29] Yun U., Yang D. (2020). Sinapic acid inhibits cardiac hypertrophy via activation of mitochondrial Sirt3/SOD2 signaling in neonatal rat cardiomyocytes. *Antioxidants*.

[30] Wu K. K. L., Long K., Lin H. (2021). The APPL1-Rab5 axis restricts NLRP3 inflammasome activation through early endosomal-dependent mitophagy in macrophages. *Nature Communications*.

[31] Khan M., Alouffi S., Khan M., Husain F., Akhter F., Ahmad S. (2020). The neoepitopes on methylglyoxal (MG) glycated LDL create autoimmune response; autoimmunity detection in T2DM patients with varying disease duration. *Cellular Immunology*.

[32] Le Bagge S., Fotheringham A., Leung S., Forbes J. (2020). Targeting the receptor for advanced glycation end products (RAGE) in type 1 diabetes. *Medicinal Research Reviews*.

[33] Koka S., Xia M., Chen Y. (2017). Endothelial NLRP3 inflammasome activation and arterial neointima formation associated with acid sphingomyelinase during hypercholesterolemia. *Redox Biology*.

[34] Deng X., Huang W., Peng J. (2018). Irisin alleviates advanced glycation end products-induced inflammation and endothelial dysfunction via inhibiting ROS-NLRP3 inflammasome signaling. *Inflammation*.

[35] Wang F., Kuo C., Ko J. (2020). Irisin mitigates oxidative stress, chondrocyte dysfunction and osteoarthritis development through regulating mitochondrial integrity and autophagy. *Antioxidants*.

[36] Pesce M., Ballerini P., Paolucci T., Puca I., Farzaei M., Patruno A. (2020). Irisin and autophagy: first update. *International Journal of Molecular Sciences*.

[37] Colaianni G., Errede M., Sanesi L. (2021). Irisin correlates positively with BMD in a cohort of older adult patients and downregulates the senescent marker p21 in osteoblasts. *Journal of Bone and Mineral Research*.

[38] Ye W., Wang J., Lin D., Ding Z. (2020). The immunomodulatory role of irisin on osteogenesis via AMPK-mediated macrophage polarization. *International Journal of Biological Macromolecules*.

[39] Gao J., Feng Z., Wang X. (2018). SIRT3/SOD2 maintains osteoblast differentiation and bone formation by regulating mitochondrial stress. *Cell Death and Differentiation*.

[40] Kim H., Lee Y., Kim H., Lee Z., Kim H. (2017). SOD2 and Sirt3 control osteoclastogenesis by regulating mitochondrial ROS. *Journal of Bone and Mineral Research*.

[41] Chen L., Wang B. Z., Xie J. (2021). Therapeutic effect of SIRT3 on glucocorticoid-induced osteonecrosis of the femoral head via intracellular oxidative suppression. *Free Radical Biology & Medicine*.

[42] Ye G., Xie Z., Zeng H. (2020). Oxidative stress-mediated mitochondrial dysfunction facilitates mesenchymal stem cell senescence in ankylosing spondylitis. *Cell Death & Disease*.

[43] Yu W., Gao B., Li N. (2017). Sirt3 deficiency exacerbates diabetic cardiac dysfunction: role of Foxo3A-Parkin-mediated mitophagy. *Biochimica et Biophysica Acta - Molecular Basis of Disease*.

[44] He X., Zeng H., Chen J. (2019). Emerging role of SIRT3 in endothelial metabolism, angiogenesis, and cardiovascular disease. *Journal of Cellular Physiology*.

[45] Ren T., Zhang H., Wang J. (2017). MCU-dependent mitochondrial Ca^2+^ inhibits NAD^+^/SIRT3/SOD2 pathway to promote ROS production and metastasis of HCC cells. *Oncogene*.

[46] Zhou X., Chen M., Zeng X. (2014). Resveratrol regulates mitochondrial reactive oxygen species homeostasis through Sirt3 signaling pathway in human vascular endothelial cells. *Cell Death & Disease*.

[47] Kerr J., Adriaanse B., Greig N. (2017). Mitophagy and Alzheimer's disease: cellular and molecular mechanisms. *Trends in Neurosciences*.

[48] Bravo-San Pedro J., Kroemer G., Galluzzi L. (2017). Autophagy and mitophagy in cardiovascular disease. *Circulation Research*.

[49] Naik P., Birbrair A., Bhutia S. (2019). Mitophagy-driven metabolic switch reprograms stem cell fate. *Cellular and Molecular Life Sciences*.

[50] Mizushima N., Komatsu M. (2011). Autophagy: renovation of cells and tissues. *Cell*.

[51] Jin M., Liu X., Klionsky D. J. (2013). SnapShot: Selective Autophagy. *Cell*.

[52] Anding A. L., Baehrecke E. H. (2017). Cleaning house: selective autophagy of organelles. *Developmental Cell*.

[53] Ren-Qi Y., Chao R., Zhao-Fan X., Yong-Ming Y. (2021). Organelle-specific autophagy in inflammatory diseases: a potential therapeutic target underlying the quality control of multiple organelles. *Autophagy*.

[54] Gao J., Liu S., Xu F. (2018). Trilobatin protects against oxidative injury in neuronal PC12 cells through regulating mitochondrial ROS homeostasis mediated by AMPK/Nrf2/Sirt3 signaling pathway. *Frontiers in Molecular Neuroscience*.

[55] Sun K., Jing X., Guo J., Yao X., Guo F. (2021). Mitophagy in degenerative joint diseases. *Autophagy*.

[56] Liu D., Ma Z., Di S. (2018). AMPK/PGC1*α* activation by melatonin attenuates acute doxorubicin cardiotoxicity *via* alleviating mitochondrial oxidative damage and apoptosis. *Free Radical Biology & Medicine*.

[57] Ye L., Fan Z., Yu B. (2012). Histone demethylases KDM4B and KDM6B promotes osteogenic differentiation of human MSCs. *Cell Stem Cell*.

[58] Golpanian S., Wolf A., Hatzistergos K., Hare J. (2016). Rebuilding the damaged heart: mesenchymal stem cells, cell-based therapy, and engineered heart tissue. *Physiological Reviews*.

[59] Harrell C., Fellabaum C., Jovicic N., Djonov V., Arsenijevic N., Volarevic V. (2019). Molecular mechanisms responsible for therapeutic potential of mesenchymal stem cell-derived secretome. *Cell*.

[60] Albrecht E., Schering L., Buck F. (2020). Irisin: still chasing shadows. *Molecular Metabolism*.

[61] Kim H., Wrann C. D., Jedrychowski M. (2019). Irisin mediates effects on bone and fat via *α*V integrin receptors. *Cell*.

