# Supplementary figures and images for: Irisin Promotes Osteogenesis by Modulating Oxidative Stress and Mitophagy through SIRT3 Signaling under Diabetic Conditions
Source: Oxid Med Cell Longev. 2022 Oct 10;2022:3319056. doi: 10.1155/2022/3319056 (PMC9576424; doi:10.1155/2022/3319056)

**Supplementary Materials:**

1. FIGURE S1

**
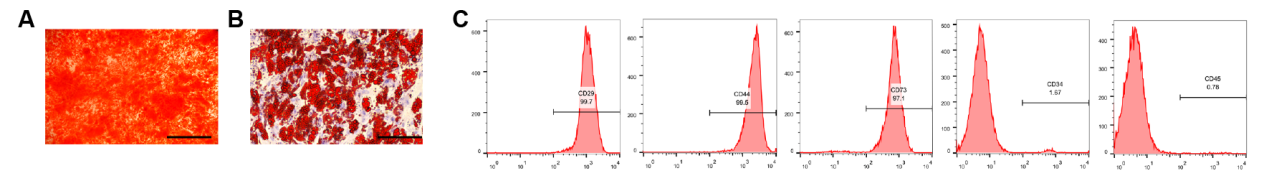
**

1. FIGURE S2

**
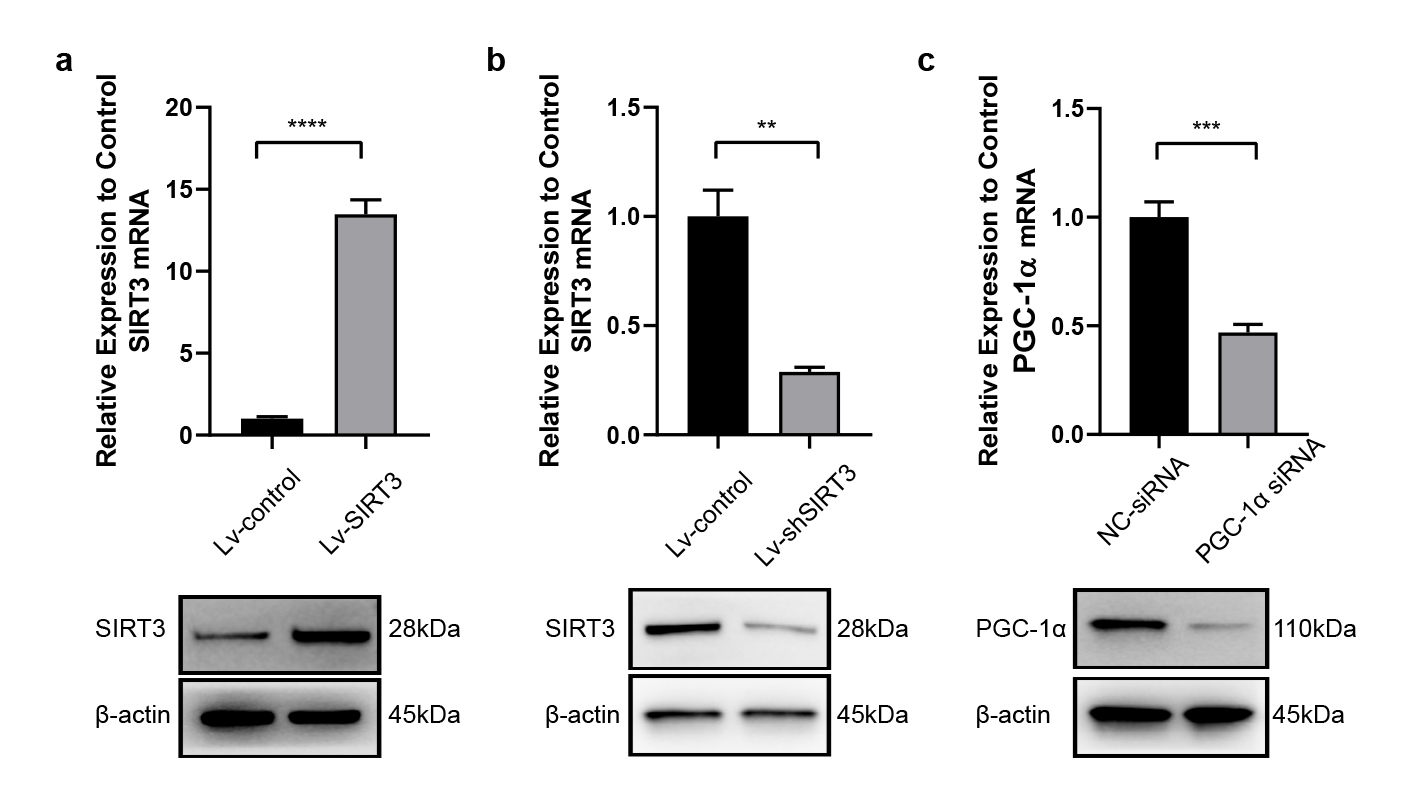
**

Supplement: Supplementary Materials — Figure S1: Identification of ASCs in vitro. (a) Assessment of the osteogenic potential of ASCs via ARS on day 21 (scale bar = 400 μm). (b) Assessment of adipogenic induction in ASCs via oil red O staining on day 14. (c) ASCs were positive for MSC markers (CD29, CD44, and CD73) but negative for hematopoietic markers (CD34 and CD45). S2: overexpression and knockdown transfection efficiencies for SIRT3 and PGC-1α. Overexpression transfection efficiencies by Lv-SIRT3 (a) and knockdown transfection efficiencies by Lv-shSIRT3 (b) and by PGC-1α-siRNA (c) were detected by qPCR (upper panel) and WB (lower panel). ∗P < 0.05, ∗∗P < 0.01, ∗∗∗P < 0.001, and ∗∗∗∗P < 0.0001. [file 3319056.f1.docx]
